# The Magnitude and Duration of *Clostridium difficile* Infection Risk Associated with Antibiotic Therapy: A Hospital Cohort Study

**DOI:** 10.1371/journal.pone.0105454

**Published:** 2014-08-26

**Authors:** Kevin A. Brown, David N. Fisman, Rahim Moineddin, Nick Daneman

**Affiliations:** 1 Division of Epidemiology, Dalla Lana School of Public Health, University of Toronto, Toronto, Ontario, Canada; 2 Department of Family and Community Medicine, University of Toronto, Toronto, Ontario, Canada; 3 Division of Infectious Diseases, Department of Medicine, Sunnybrook Health Sciences Centre, University of Toronto, Toronto, Ontario, Canada; The University of Tokyo, Japan

## Abstract

Antibiotic therapy is the principal risk factor for *Clostridium difficile* infection (CDI), but little is known about how risks cumulate over the course of therapy and abate after cessation. We prospectively identified CDI cases among adults hospitalized at a tertiary hospital between June 2010 and May 2012. Poisson regression models included covariates for time since admission, age, hospitalization history, disease pressure, and intensive care unit stay. Impacts of antibiotic use through time were modeled using 4 measures: current antibiotic receipt, time since most recent receipt, time since first receipt during a hospitalization, and duration of receipt. Over the 24-month study period, we identified 127 patients with new onset nosocomial CDI (incidence rate per 10,000 patient days [IR] = 5.86). Of the 4 measures, time since most recent receipt was the strongest independent predictor of CDI incidence. Relative to patients with no prior receipt of antibiotics in the last 30 days (IR = 2.95), the incidence rate of CDI was 2.41 times higher (95% confidence interval [CI] 1.41, 4.13) during antibiotic receipt and 2.16 times higher when patients had receipt in the prior 1–5 days (CI 1.17, 4.00). The incidence rates of CDI following 1–3, 4–6 and 7–11 days of antibiotic exposure were 1.60 (CI 0.85, 3.03), 2.27 (CI 1.24, 4.16) and 2.10 (CI 1.12, 3.94) times higher compared to no prior receipt. These findings are consistent with studies showing higher risk associated with longer antibiotic use in hospitalized patients, but suggest that the duration of increased risk is shorter than previously thought.

## Introduction

*Clostridium difficile* infection (CDI) is a hospital and community-acquired disease that especially impacts elderly hospitalized patients receiving antibiotics [1]. CDI incidence in North American hospitals has increased drastically in the last 30 years, and this has been hypothesized to be due to the emergence of new, hyper-virulent strains and to the ubiquity of antibiotic use among hospitalized patients [2]. In temperate countries, CDI has a seasonality that follows, with several months delay, that of seasonal prescribing of broad spectrum antibiotics and of pneumonia [3]–[5].

Antibiotic receipt represents the most important known risk factor for CDI; it is thought to induce CDI risk by denuding the gut of protective bacteria and increasing *C. difficile* spore germination [6], [7]. Almost all classes of antibiotics have been found to increase CDI risk; certain classes including fluoroquinolones, cephalosporins and clindamycin are thought to have a more potent impact while others, such as tetracyclines, may not change CDI risk at all [8]. Several studies have followed patients for periods of 30 to 100 days post-admission and shown that a relatively elevated incidence of CDI exists for patients post-discharge [9]–[12]. Recent studies have used survival analysis incorporating time-varying antibiotic exposures and indicated that increased duration, number, and dosages of antibiotics were associated with increased risk [13], [14]. Weighted cumulative exposure models build on survival analysis and stipulate that current risks may be considered as functions of past exposures [15], and may be used to explicitly and flexibly estimate the day-by-day CDI risk during and after antibiotic therapy. As such, our objective was to assess the degree to which risks associated with antimicrobial exposures both cumulate over the course of antimicrobial therapy and abate after cessation.

## Methods

### Ethics Statement

Study approval was obtained from the Research Ethics Board of Sunnybrook Health Sciences Centre who waived the need for patient consent because there was no contact with the patients and anonymity was assured.

### Study Design and Participants

A cohort study design was used to assess the association of antibiotic exposure with the incidence of CDI among patients admitted to Sunnybrook hospital, a large acute care teaching hospital located in Toronto, Canada. The source cohort consisted of all patients over 18 years old, without a previous CDI diagnosis, and hospitalized in an acute care ward at Sunnybrook hospital in the June 1, 2010 to May 31, 2012 period and excluded time spent in the hospital's psychiatry ward.

### CDI Case Definition

*C. difficile* infected patients were prospectively identified by the Infection Prevention and Control (IPC) department via active surveillance during the study period. In accordance with the provincial surveillance definition [16], a CDI case was defined as any hospitalized patient with either: (a) laboratory confirmation of a positive toxin assay together with diarrhea, or (b) visualization of pseudomembranes on sigmoidoscopy, colonoscopy, or histopathology. For the purposes of surveillance, diarrhea was defined as two or more loose/watery bowel movements in a 24-hour period, which was unusual or different for the patient, and with no other recognized etiology. Among patients developing CDI, days after the first CDI infection were excluded from the at-risk set. Time at risk was restricted to that of hospitalized patients up until the beginning of symptoms of the first disease onset and excluded the first two days of patients' hospital admissions (patients without a hospital exposure within the previous 4 weeks cannot be considered to have nosocomial acquisition in their first two days of admission according to the provincial CDI definition). Toxin assays at the hospital have been performed by polymerase chain reaction (PCR) since September 2009, which includes the entire study period.

For CDI cases, event time was the number of days from hospital admission to symptom onset, or positive toxin assay for rare cases in which symptom onset was missing. For non-cases, censoring time was the number of days from hospital entry until discharge, study termination, or death.

### Antimicrobial Exposure Assessment

Patient antibiotic exposures were drawn from pharmacy dispensing records. We examined for daily receipt of any antibiotic but excluded exposure to metronidazole and oral vancomycin since these may be treatments for CDI. Daily antibiotic receipt was classified according to the Anatomical Therapeutic Chemical (ATC) Classification System, 17^th^ edition [17]. Only antibiotic classes with a prevalence of at least 20 per 1,000 patient-days of follow-up (2%) were analyzed individually; preliminary analyses identified penicillins (ATC: J01C), cephalosporins/carbapenems (J01D), and fluoroquinolones (J01M) as meeting this criterion. We further subdivided penicillins into broad (J01CA, J01CG and J01CR) and narrow spectrum agents (J01CE and J01CF) and cephalosporins/carbapenems into 1st and 2nd generation cephalosporins (J01DB and J01DC), 3rd and 4th generation cephalosporins (J01DD and J01DE), and carbapenems (J01DH), for a total of 8 antibiotic classes that were analyzed individually. We also identified daily receipt of the following 6 infrequently prescribed classes of antibiotics: tetracyclines (J01A), nitrofurantoins (J01XE), sulfanomides and trimethoprim (J01E), macrolides and streptogramins (J01FA and J01FG), aminoglycosides (J01G), and lastly, clindamycin and other lincosamides (J01FF).

Using the antibiotic classes identified above, we developed two alternative measures of antibiotic exposure: (1) an index representing the number of distinct classes of antibiotics a patient received on a given day (classified as 0, 1 or ≥2), and (2) a categorical antimicrobial risk index based on Brown et al. [18] which classified patients according to whether they received a high risk antibiotic (defined as receipt of cephalosporins/carbapenems, fluoroquinolones, or clindamycin and other lincosamides), had received a medium risk antibiotic but not a high risk antibiotic (defined as penicillins, sulfanomides and trimethoprim, macrolides and streptogramins, or aminoglycosides), or had received no antibiotics or a low risk antibiotic only (defined as receipt of tetracylines).

### Modeling Time-Varying Antimicrobial Exposures

We created 4 variables based on patients' unique antibiotic exposure histories: (1) current antibiotic receipt, which was dummy coded as 1 for days when a patient received an antibiotic, and 0 otherwise, (2) the time lapse since the most recent antibiotic use (in days), which was categorized as 0 (current use), 1–5, 6–30, >30 (including no use), (3) time lapse since the start of the first antibiotic exposure during a given hospitalization, which was categorized as 0–2, 3–6, 7–14, 15–30 and >30 (including no use), and (4) the duration of antibiotic use, categorized as 0 (no use), 1–3, 4–6, 7–11 and >11.

### Other Risk Factors

Patient age, sex, hospital pharmacy record, and bed assignment, were obtained from electronic hospital administrative records. Infection pressure was derived by calculating the number of diagnosed infectious patients with CDI in each ward of the hospital at noon of each day using patient location records. The infectious period of a given diagnosed patient (which included cases and non-cases with non-nosocomial or recurrent disease) was defined as starting on the day *after* symptom onset to 14 days after the positive test associated with case detection. This is in keeping with Dubberke et al. [19] with the exception that the day of onset was excluded from disease pressure calculations so that new cases did not contribute disease pressure risk to themselves. We also measured the use of antacids (ATC: A02), laxatives (A06), feeding tube [20], and whether a patient had stayed in an intensive care unit (ICU).

### Statistical Analysis

For bivariate analyses in which we compared characteristics of the at-risk period of cases with a 10% simple random sample of non-case patients (controls), two-sided p-values were assessed with Pearson's chi-squared test for categorical variables and with the Wilcoxon rank-sum test for continuous variables. For the principal case-cohort analysis based on Poisson regression (models described below), which were used to measure unadjusted incidence rates and unadjusted and adjusted incidence ratios, we weighted all of the control patients' follow-up times by 10 and all of the case patients' follow up times by 1, so as to reflect rates from the original, complete, cohort, as per the Barlow method [21].

To estimate the impact of antibiotic exposures on CDI risk, we developed weighted Poisson regression models that aimed to predict the time elapsed from hospital admission to the occurrence of a first CDI infection. Our data was structured in counting process format with one record for each patient-day. For each of the 4 antibiotic exposure covariates, two models were fitted to the data. The 4 unadjusted models included no covariates other than antibiotic exposures; incidence rate ratios were estimated using Poisson regression. The 4 adjusted models included 6 potential confounders: time since admission (modeled as a b-spline with knots at 5 and 15 days), patient age (classified as <45, 45 to <65, ≥65 years), sex, number of previous hospital admissions (classified as 0, 1, ≥2), patient-days of infection pressure in the past 10 days, and ICU admission in the past 10 days. The number of adjustment factors was restricted in order to ensure at least 10 events per covariate [22], and the selection of covariates was based on established associations with CDI risk [13], [23]. For the adjusted models, intra-patient correlation was accounted-for using generalized estimating equations [24]. Statistical fit for all models was assessed using Akaike's Information Criterion [25].

Subsequently, we used the best fitting of the 4 antibiotic exposure covariates to determine risk associated with the two risk indexes and for each of the 8 antibiotic classes. For unadjusted and adjusted estimates of the antibiotic-specific risks, antibiotic exposure adjustment variables were derived which measured exposure to any other antibiotic without exposure to the antibiotic in question.

Analyses were conducted using R statistical software (v3.0.2); the glm and geeglm [26] functions were used for the unadjusted and adjusted statistical models, respectively. R statistical software analysis code is provided in Appendix S1.

### Sensitivity Analyses

In order to assess the potential impact of uncertainty of diagnostic timing on the analyses, we conducted a sensitivity analysis using positive *C. difficile* PCR test date rather than symptom onset date to define the outcome timing. Also, because pre-admission antibiotic exposure information was not available, we conducted an additional sensitivity analysis excluding cases and patient time in the first 10 days of each hospitalization to investigate the impact of exposure history incompleteness.

## Results

### Description of Cohort

**Figure 1:**
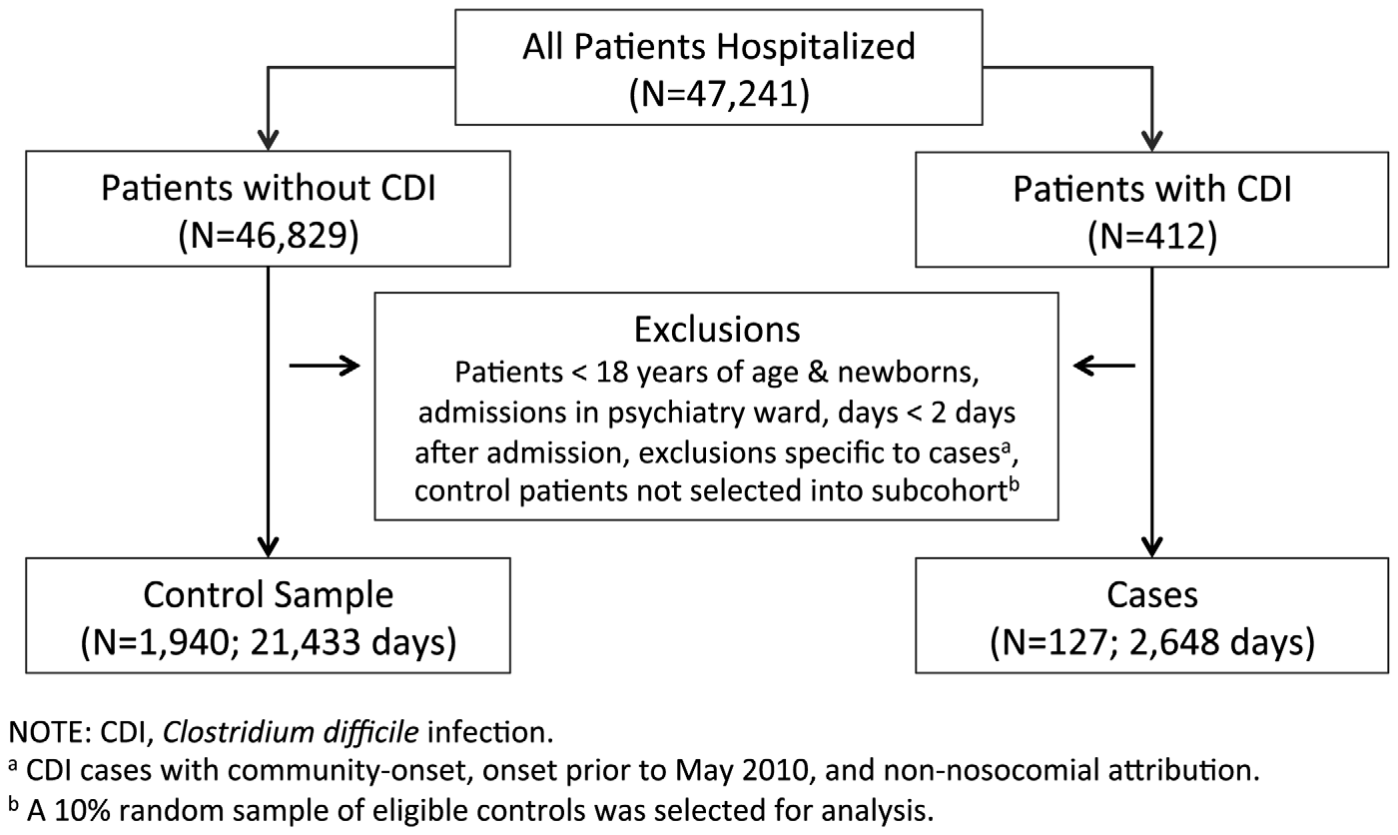
Patients and Patient-Time Included in the Final Cohort, Sunnybrook Hospital, Toronto, Canada, June 2010 to May 2012.

Over the two-year study period, and before exclusion of ineligible patients, a total of 47,241 patients were identified as having been admitted to Sunnybrook Health Science Centre (Figure 1). Of these, 412 were diagnosed with CDI; after exclusion of patients with recurrent or non-nosocomial CDI, and patients with onset of CDI while out of hospital, or within the first two days of an admission, 127 nosocomial case patients remained. After removal of ineligible non-case patients, the 10% control cohort selection consisted of 1,940 patients. The incidence of CDI in the cohort was 5.86 per 10,000 days of follow-up (127/216,978).

### Demographic and Exposure Characteristics of Cases and Controls

The median age of cases (72.0) was almost 5 years older than that of controls (67.3, p = 0.14, Table 1). Cases had a higher rate of exposure to other symptomatic CDI patients in their own ward (median, 20 per 100 person-days versus 0 per 100 person-days, p = 0.12). About half (50.4%) of case patients spent time in the ICU during their risk period compared to 19.5% of controls (p<0.001).

**Table 1:** Selected Characteristics of Case and Control Patients, Sunnybrook Hospital, Toronto, Canada, June 2010 to May 2012.

	Incident Cases	Controls	*P* value
	(N = 127)	(N = 1940)	
	N (%)	N (%)	
Age, median (IQR), y	72.0 (57.6–79.8)	67.3 (53.2–79.2)	0.14
Male sex	69 (54.3)	989 (51.0)	0.52
Admissions			
1	76 (59.8)	1494 (77.0)	<0.001
2	30 (23.6)	302 (15.6)	<0.001
≥3	21 (16.5)	144 (7.4)	<0.001
ICU stay	64 (50.4)	379 (19.5)	<0.001
Disease pressure^a^			
median per 100 patient-days (IQR)	20.0 (0.0–50.0)	0.0 (0.0–50.0)	0.12
Days of antibiotic exposure,			
median per 100 patient-days (IQR)	46.2 (25.0–76.0)	0.0 (0.0–60.0)	<0.001
Antibiotic exposure			
Total	110 (86.6)	961 (49.5)	<0.001
Penicillins	38 (29.9)	220 (11.3)	<0.001
Broad-spectrum	29 (22.8)	191 (9.8)	<0.001
Narrow-spectrum	12 (9.4)	43 (2.2)	<0.001
Cephalosporins/carbapenems	91 (71.7)	642 (33.1)	<0.001
1^st^ & 2^nd^ generation	44 (34.6)	388 (20.0)	<0.001
3^rd^ & 4^th^ generation	59 (46.5)	326 (16.8)	<0.001
Carbapenems	11 (8.7)	41 (2.1)	<0.001
Fluoroquinolones	49 (38.6)	381 (19.6)	<0.001
IV vancomycin	28 (22.0)	107 (5.5)	<0.001
Other exposures			
Antacids (PPIs and H2 inhibitors)	96 (75.6)	1268 (65.4)	0.024
Laxatives	91 (71.7)	1187 (61.2)	0.024
Feeding tube	53 (41.7)	263 (13.6)	<0.001

Abbreviations: PPI, proton pump inhibitor; ICU, intensive care unit; IQR, interquartile range.

a equal to the number of patients diagnosed with CDI in the same ward as a given patient each day.

b 2 degree of freedom Pearson's Chi-square test.

A larger proportion of case patients received antibiotics during their risk period relative to controls (86.6% vs 49.5%, p<0.001). The majority (71.7%) of cases received a cephalosporin class of antibiotic compared to 33.1% of controls (p<0.001). Similarly, penicillins and fluoroquinolones were more likely to be prescribed among case patients compared to controls. Among case patients, median symptom onset date was 9 days after admission (IQR: 5–17 days).

### Risk Associated with Antibiotic Exposures

The incidence of CDI when patients received antibiotics was 8.43 per 10,000 days (64/75,959) compared to 4.47 (63/141,019) when patients did not receive antibiotics (Table 2); it follows that the incidence rate ratio (IRR) associated with current antibiotic exposure was 1.89 (95% confidence interval [CI] 1.33, 2.67). Adjustment for time since admission, patient age, sex, number of previous hospital admissions, disease pressure, and current or prior ICU admission reduced the IRR slightly, to 1.79 (95% CI 1.24, 2.59).

**Table 2:** Timing and Magnitude of CDI Risk Associated with Antibiotic Exposures.

				Unadjusted		Adjusted^c^	
	CDI Cases	Follow-up	IR^a^	IRR	Δ AIC^b^	IRR	Δ AIC^b^
	(N)	(days)		(95% CI)		(95% CI)	
Antibiotic use on current day							
No	63	141019	4.47	Reference		Reference	
Yes	64	75959	8.43	1.89 (1.33, 2.67)	0	1.79 (1.24, 2.59)	0
Time since end antibiotic therapy (d)							
0 (current receipt)	64	75959	8.43	2.86 (1.73, 4.72)		2.41 (1.41, 4.13)	
1–5	30	36950	8.12	2.75 (1.56, 4.85)		2.16 (1.17, 4.00)	
6–30	13	36288	3.58	1.21 (0.60, 2.44)		0.98 (0.48, 2.00)	
>30, or no antibiotic use	20	67781	2.95	Reference	−9.5	Reference	−4.6
Time since start of first antibiotic (d)							
0–2	7	21060	3.32	1.08 (0.47, 2.49)		1.39 (0.56, 3.46)	
3–6	40	38007	10.52	3.43 (2.11, 5.59)		3.10 (1.73, 5.54)	
7–14	33	37530	8.79	2.87 (1.72, 4.77)		1.76 (0.98, 3.13)	
15–30	20	32313	6.19	2.02 (1.13, 3.60)		1.56 (0.84, 2.90)	
>30, or no antibiotic use	27	88068	3.07	Reference	−14.6	Reference	−0.7
Cumulative duration of antibiotic use (d)							
0 (no prior receipt)	20	67781	2.95	Reference		Reference	
1–3	25	45201	5.53	1.87 (1.04, 3.37)		1.60 (0.85, 3.03)	
4–6	28	34596	8.09	2.74 (1.55, 4.87)		2.27 (1.24, 4.16)	
7–11	28	35968	7.78	2.64 (1.49, 4.68)		2.10 (1.12, 3.94)	
>11	26	33432	7.78	2.64 (1.47, 4.72)	−0.07	2.84 (1.39, 5.81)	5.1

Abbreviations: AIC, Akaike's Information Criterion; CDI, *Clostridium difficile* infection; CI, confidence interval; d, days; IR, incidence rate; IRR, incidence rate ratio.

a Incidence rate, per 10,000 patient-days.

b The difference in AIC relative to the reference model (current antibiotic use): negative numbers denote an improvement in fit. Δ AIC <−2 was considered a statistically significant improvement in fit at p<0.05.

c Adjusted for time since hospital admission, age, sex, number of previous hospital admissions, infection pressure, and current or prior ICU admission.

**Figure 2:**
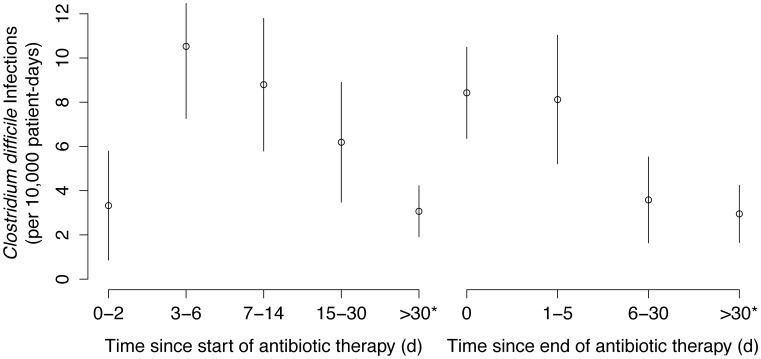
The Magnitude and Duration of *Clostridium difficile* Infection Risk After Antibiotic Therapy, Sunnybrook Hospital, Toronto, Canada, June 2010 to May 2012. Among inpatients, the incidence of *Clostridium difficile* infection was highest in the period 3 to 14 days after the start of antibiotic therapy, during antibiotic therapy, and within 5 days of the end of antibiotic therapy. * Includes patients without any identified antibiotic use.

Among the 4 antibiotic exposure measures, the time since last antibiotic use was the most important independent predictor of CDI onset, and yielded a statistically significant improvement in the prediction of CDI onset (Δ adjusted AIC = −4.6). The incidence of CDI (per 10,000 days) was 2.95 when patients had received no antibiotics in the previous 30 days (reference), 8.43 when patients were currently receiving antibiotics (adjusted IRR: 2.41, 95% CI 1.41, 4.13), 8.12 when patients had recent receipt in the last 1–5 days (adjusted IRR: 2.16, 95% CI 1.19, 3.32), and 3.58 when patients had received antibiotics in the last 6–30 days (adjusted IRR: 0.98, 95% CI 0.48, 2.00, Figure 2).

The time elapsed since the start of the first antibiotic exposure in a given hospitalization also yielded an improved prediction of the timing of CDI onset (Δ adjusted AIC = −0.7). The observed association was highest 3–6 days (adjusted IRR = 3.10, 95% CI 1.73–5.54) and 7–14 days (adjusted IRR = 1.76, 95% CI 0.98–3.13) after the start of antibiotics. The duration of antibiotic exposure yielded a worse model fit relative to the adjusted model with current antibiotic use (Δ adjusted AIC = 5.1).

### Sensitivity Analyses

For all remaining analyses including the sensitivity analyses and the investigation of antibiotic class-specific effects, we categorized antibiotic exposure history as receipt of antibiotics in the last 5 days (which included current receipt), or no receipt in the last 5 days. The adjusted risk was 2.35 (95% CI 1.53, 3.60) for patients that had received an antibiotic in the previous 5 days (Table 3). We conducted 2 different sensitivity analyses; neither impacted the estimated risk substantially. In the first sensitivity analysis, we considered the impact of restricting the dataset to follow-up ≥10 days after admission, so that complete information on antibiotic history was known for a larger proportion of patients. The adjusted IRR for risk extended 5 days beyond the end of antibiotic use was similar to that of the full cohort (2.23, 95% CI 1.21, 4.13). We also considered a sensitivity analysis in which we varied the assignment of CDI event date; when positive test date rather than symptom onset was used to define the outcome, the number of eligible cases increased from 127 to 130. The adjusted incidence rate for antibiotic use in the last 5 days was similar, at 2.79 (95% CI 1.47, 5.27).

**Table 3:** CDI Risk Associated with Antimicrobial Exposures During and Within 5 days of the End of Antimicrobial Therapy, for Antibiotic Risk Indexes and Specific Antibiotic Exposures.

				Unadjusted		Adjusted^c^	
Exposure in the preceding 5d	CDI Cases	Follow-up	IR^a^	IR Ratio	Δ AIC^b^	IR Ratio	Δ AIC^b^
	(N)	(days)		(95% CI)		(95% CI)	
Any antibiotic							
No	33	104069	3.2	Reference		Reference	
Yes	94	112909	8.3	2.63 (1.77, 3.90)	0	2.35 (1.53, 3.60)	0
Number of antibiotics							
0	33	104069	3.2	Reference		Reference	
1	64	73593	8.7	2.74 (1.80, 4.17)		2.49 (1.59, 3.92)	
≥2	30	39316	7.6	2.41 (1.47, 3.95)	1.6	2.09 (1.23, 3.55)	1.6
Antibiotic risk index							
None or low-risk	35	106396	3.3	Reference		Reference	
Medium-risk	12	19256	6.2	1.89 (1.33, 2.67)		1.79 (1.24, 2.59)	
High-risk	80	91326	8.8	2.66 (1.79, 3.96)	2.1	2.43 (1.59, 3.74)	1.3
Class of antibiotic^d^							
Penicillins	24	25103	9.5	3.02 (1.78, 5.10)	1.4	2.77 (1.56, 4.90)	1.7
Broad-spectrum	16	18876	8.5	2.67 (1.47, 4.86)	2.0	2.39 (1.26, 4.53)	2.3
Narrow-spectrum	8	6857	11.7	3.68 (1.70, 7.97)	1.1	3.64 (1.47, 9.00)	1.5
Cephalosporins	61	66330	9.2	2.90 (1.90, 4.43)	0.5	2.70 (1.69, 4.32)	0.2
1^st^ & 2^nd^ generation	31	38508	8.0	2.54 (1.55, 4.15)	1.9	2.36 (1.36, 4.10)	2.3
3^rd^ & 4^th^ generation	32	27092	11.8	3.72 (2.29, 6.06)	−2.8	3.40 (2.02, 5.72)	−2.5
Carbapenems	6	5962	10.1	3.17 (1.33, 7.57)	1.8	2.40 (0.93, 6.23)	2.3
Fluoroquinolones	24	30286	7.9	2.50 (1.48, 4.23)	1.9	2.16 (1.26, 3.72)	2.0
IV vancomycin	16	11883	13.5	4.25 (2.34, 7.71)	−1.6	3.19 (1.62, 6.26)	1.2

Abbreviations: AIC, Akaike's Information Criterion; CDI, *Clostridium difficile* infection; CI, confidence interval; d, days; IR, incidence rate; IRR, incidence rate ratio.

a Incidence rate, per 10,000 patient-days.

b The difference in AIC relative to the reference model (receipt of any antibiotic in the previous 5 days): negative numbers denote an improvement in fit. Δ AIC <−2 was considered a statistically significant improvement in fit at p<0.05.

c Adjusted for time since hospital admission, age, sex, number of previous hospital admissions, infection pressure, and current or prior ICU admission.

d Each antibiotic group was assessed in a separate model; the reference group for each model was no receipt of antibiotics in the last 5 days.

### Antibiotic Risk According to Antimicrobial Classes

In order to consider differences in the level of risk among antibiotic users, we considered risk among patients with exposure to various combinations of antibiotics and to specific antibiotics (Table 3). The adjusted risk associated with CDI was similar when patients either received a single class of antibiotic (IRR = 2.49, 95% CI 1.59, 3.92) or when patients received multiple classes of antibiotics (IRR = 2.09, 95%CI 1.23, 3.55). For our antibiotic risk index variable, which was based on established associations of antibiotics with CDI risk, the adjusted risk was 1.79, (95%CI 1.24, 2.59) for medium-risk antibiotics and was 2.43 (95%CI 1.59, 3.74) for high-risk antibiotics.

For each of the 8 antibiotic classes and subclasses for which there was sufficient exposure for individual analysis (>2% of subcohort patient-time), we measured the risk of the given antibiotic taken alone or in combination with other antibiotics, relative to no antibiotic exposure. In adjusted analyses, all 3 of the most prescribed antimicrobial classes demonstrated similarly large hazard ratios. Of the subclasses, 3^rd^ & 4^th^ generation cephalosporins taken alone or in combination, had higher risk in comparison to other antibiotic classes (adjusted IRR = 3.40, 95%CI 2.02, 5.72).

## Discussion

Our observational study of 127 CDI cases and 2 years of follow-up on patients at a large tertiary hospital found that: (1) in-hospital CDI risk was highest 3 to 14 days after the start of the first antibiotic course, (2) elevated CDI risk persisted for a period of 5 days after the end of antimicrobial therapy, and (3) patients with longer antibiotic courses were at higher risk of developing CDI than patients with shorter courses, but even short courses and single doses of antibiotics incur a substantial risk of inducing CDI.

Our study is the first to compare the duration of antibiotic use, time since antibiotic initiation, and time since antibiotic cessation as measures of CDI risk; our results showed that measuring the time since last antibiotic use is the most predictive metric for quantifying risk for a patient, whereby risk during and within 5 days of cessation was the most elevated. Antibiotic duration has already been shown to be more predictive than cumulative dosage for predicting CDI risk [13]; this study goes one step further and shows that time since cessation of antibiotics is a better predictor than antibiotic duration. Since colonization resistance is thought to be greatly diminished both during and for a period after the end of antibiotic use, our findings support the importance of this mechanism and reflect findings from animal and in vitro models of colonization resistance as it relates to CDI [27], [28]. However, our study demonstrated substantially shorter impacts of antibiotic use on patient risk compared to previous empirical studies: in a case-control study of 337 patients with healthcare-associated CDI, risk was found to be relatively constant both during antibiotic use and for a period of 30 days after cessation [29], diminishing more than 30 days after the end of antimicrobial exposure. Our study population was restricted to inpatients that may have developed CDI more rapidly than outpatients and patients discharged from hospital. Lastly, although our findings show that risk is principally driven by time since antibiotic cessation, inferring from the low risk for 2 days after the start of antimicrobial use, our models provide empirical evidence that the incubation period of CDI is at least 48h [30].

A strength of this study was our use of a combination of analytic methods tailored to the study of nosocomial infections. Foundational texts in epidemiologic methods principally covered the use of logistic regression and survival analysis for the study of chronic diseases that develop over the course of many years [31], [32]. As such, some of these methods may not be optimal for understanding nosocomial infections that develop rapidly over the course of days and hours from the time of hospital admission, and are driven by contagion [33], [34]. In order to address these characteristics of nosocomial infections, our analyses were based on an extension of survival analysis that incorporated both weighted cumulative exposures to account for the time-sensitive nature of antibiotic effects [15], and disease pressure, to incorporate the impacts of contagion from patients with CDI [35]. Future analyses of infectious diseases transmitted by the fecal-oral route could attempt to quantify ingestion of disease-causing organisms [36]. Ignoring contagion not only leads to incorrect estimation of risk factors due to residual confounding and unaccounted spatial clustering of cases, but also appears to have driven an unbalanced understanding of CDI etiology focusing excessively on intrinsic risk factors. Regarding this, although antibiotics are well established as the principal risk factor for CDI acquisition [18], [37], a recent study was unable to identify CDI transmission sources for 45% of cases [38].

A limitation of our study was our lack of information on outpatient antimicrobial exposures prior to patient hospitalization, since our exposure free reference group used for calculating hazard ratios could have included patients exposed to antibiotics prior to admission. However, our findings were robust in sensitivity analyses considering subsets of patients with more prolonged hospitalization and therefore more complete antibiotic exposure histories. Further, our study lacked information on post-discharge *C. difficile* diagnoses. Considering discharged patients as censored surmounted this limitation, but means that our results are most generalizable to acute, hospital-onset CDI. Furthermore, clinical teams are aware of antimicrobial exposures which may prompt diagnostic testing for CDI [27], and this surveillance bias may lead to an overestimation of the risk associated with current or recent antibiotic exposure. We had no data on *C. difficile* colonization status and timing of acquisition of the organism, which would be expected to influence the time lapse between antibiotic exposure and disease onset. Finally, our hospital information system lacked information on patient comorbidities and other healthcare exposures including discharges and outpatient visits to other hospitals in the region.

Antibiotic use is the most important risk factor for CDI, and a substantial amount of research has considered the risk of CDI associated with different antibiotic exposures. In this study of the association between antibiotic exposures and CDI risk in hospitalized adults, we focused on the timing of increased risk; we found that risk appears 3 days after the onset of antibiotic use, and continues for a period of 5 days after the end of antibiotics, and is relatively unimportant thereafter. Further research may consider how different antibiotics may induce different time-varying risks in order to differentiate antibiotic impacts and improve patient outcomes.

## Supporting Information

Appendix S1 **R Statistical Software Analysis Code For Tables and Poisson Regression Models.**
(TXT)
